# Relationship between maximum oxygen uptake and peripheral vasoconstriction in a cold environment

**DOI:** 10.1186/s40101-017-0158-2

**Published:** 2017-12-06

**Authors:** Takafumi Maeda

**Affiliations:** 1 Department of Human Science, Faculty of Design, Kyushu University, 4-9-1, Shiobaru, Minami-ku, Fukuoka, 815-8540 Japan; 2 Physiological Anthropology Research Center, Faculty of Design, Kyushu University, 4-9-1, Shiobaru, Minami-ku, Fukuoka, 815-8540 Japan

**Keywords:** Thermoregulation, Physical fitness, Cross-adaptation, Vasoconstriction, Metabolic heat production, Cold

## Abstract

**Background:**

Various individual characteristics affect environmental adaptability of a human. The present study evaluates the relationship between physical fitness and peripheral vasoconstriction in a cold environment.

**Methods:**

Seven healthy male students (aged 22.0 years) participated in this study. Cold exposure tests consisted of supine rest for 60 min at 28 °C followed by 90 min at 10 °C. Rectal and skin temperatures at seven sites, oxygen consumption, and the diameter of a finger vein were measured during the experiment. Metabolic heat production, skin heat conductance, and the rate of vasoconstriction were calculated. Individual maximum oxygen consumption, a direct index of aerobic fitness, was measured on the day following the cold exposure test.

**Results:**

Decreases in temperature of the hand negatively correlated with the changes in rectal temperature. Maximum oxygen consumption and the rate of vasoconstriction are positively correlated. Furthermore, pairs of the following three factors are also significantly correlated: rate of metabolic heat production, skin heat conductance, and the rate of vasoconstriction.

**Conclusion:**

The results of this study suggested that the capacity for peripheral vasoconstriction can be improved by physical exercise. Furthermore, when exposed to a cold environment, fitter individuals could maintain metabolic heat production at the resting metabolic level of a thermoneutral condition, as they correspondingly lost less heat.

## Introduction

Human thermoregulatory functions are influenced by various factors, such as genetic factors, season, lifestyles, and individual physical and physiological characteristics [1–4]. Also, aerobic exercise capacity effects thermoregulatory function, and physical endurance training improves thermal adaptability.

Several studies have investigated the effects of physical training on thermoregulation in a hot environment [5–8], and the findings have suggested that physical training improves the capacity for thermoregulation. Many investigators have found improved ability to thermoregulate by cross-adaptation to exercise-induced hyperthermia, through improvements in and enhancements of vasodilation [5–7] and the sweat response [8].

Regarding thermoregulatory ability in a cold environment, physical endurance training increases cold tolerance, and individuals with higher levels of physical fitness exhibit higher adaptability to cold [9–19]. According to such studies, training increases metabolic heat production in a cold environment, which leads to a better cold tolerance [9–11, 13, 15, 18]. However, the effects of aerobic training on the ability to inhibit heat loss in a cold environment are controversial, because studies have indicated that skin temperature in fitter individuals exposed to cold can be either higher [11, 20] or lower [16, 19].

Previous studies have used skin heat conductance as an index of heat loss, from which the degree of vasoconstriction was estimated [11, 12, 18]. Some investigators have reported that the skin heat conductance of relatively fit individuals is greater than that of less-fit individuals during cold exposure and heat loss is more substantial [11, 12]. But others have found lower skin heat conductance and less heat loss among relatively fit individuals exposed to a cold environment when both trained and untrained groups had the same ratio of body fat [18]. Thus, the relationship between physical fitness and cold-induced vasoconstriction determined from skin heat conductance is obscure and probably influenced by body fatness.

Although skin heat conductance is reflected as heat loss and calculated as the differences between the core and skin temperature and between the skin and ambient temperature, it does not directly reflect vasoconstriction. Furthermore, because physical characteristics (particularly subcutaneous fat) affect skin heat conductance, isolating only the effects of physical fitness and/or training is difficult. Thus, vasoconstriction that is an index of cold tolerance cannot be evaluated by skin heat conductance, which also explains neither vasoconstriction nor the mechanisms involved in changes or improvements in physiological adaptability conferred by physical training.

Daanen (2003) in a review of local cold tolerance among humans noted the difficulties in noninvasively and continuously measuring blood vessel diameter as an index of vasoconstriction [21]. However, the vascular diameter can now be measured noninvasively and continuously using near-infrared light in Japan, which used for a clinical investigation [22].

Aerobic training improves the compliance of peripheral blood vessels [23–26], as well as the autonomic nervous function controlling the vasomotor system [27, 28]. Therefore, we considered that peripheral vasoconstriction would be improved by physical training, and thus heat loss would be more inhibited in a cold environment.

We postulated that fitter individuals have better vasoconstriction and better heat loss inhibition in the cold. The present study focused on the peripheral vasomotor system as a key factor involved in the inhibition of heat loss in a cold environment. The first objective was to determine the correlation between physical fitness and the degree of vasoconstriction measured directly on fingers. The second objective was to estimate the effects of aerobic physical fitness on the mechanisms of thermoregulation in the context of a cold environment, and, in particular, to determine the balance between increased metabolic heat production and the inhibition of heat loss.

## Methods

### Subjects

The Ethics Committee of Fukushima Medical University approved the study protocol. The experimental test procedures were explained in detail to various individuals who then provided written informed consent to participate in the study. Seven healthy male students (age 22.0 ± 1.4 years old) volunteered to participate. All of them were healthy and belong to sports clubs such as soccer, rugby, and aikido which was conducted for 1~2 h/day and 2~3 days/week. The physical characteristics of the subjects are given in Table 1. Body surface area (BSA) was calculated from the height and weight of each participant using a formula adapted for adult Japanese males (Takahira, 1925: [BSA (cm^2^)] = [weight (kg)]^0.425^ × [height (cm)]^0.725^ × 72.76). Body fat (%) was measured on a scale using the impedance method with four electrodes (TBF-102, Tanita, Japan). Subcutaneous fat thickness was calculated as average skinfold thickness, which was measured with a caliper at the subzygomatic border, hyoid region, breast, side breast, subscapular region, abdomen, lumbar region, front and back thighs, knee, calf, and triceps. We assumed that the whole body subcutaneous fatness is related to finger subcutaneous fat, although we did not measure the finger subcutaneous fat thickness.

**Table 1:** Physical and physiological characteristics of participants

Characteristics	Mean ± SD
Age (years)	22.0 ± 1.53
Height (cm)	169.0 ± 5.20
Body mass (kg)	61.7 ± 7.12
Body fat (%)	16.6 ± 3.60
Body mass index (kg/m^2^)	21.6 ± 1.84
Body surface area (m^2^)	1.72 ± 0.11
Subcutaneous fat thickness (cm)	0.50 ± 0.17
$\overset{\cdot }{\mathrm{V}}$O_2max_ (ml/min/kg)	52.3 ± 1.94

### Cold exposure test

**Fig. 1:**
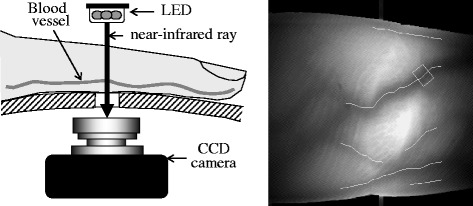
System for measuring diameter of the finger blood vessels

Rectal (*T*_re_) and skin temperatures at the forehead, abdomen, forearm, back of the hand, thigh, shin, and instep were measured at 1 min intervals using a thermistor-thermometer with a data-logger (LT-8, Gram Corp., Japan). The mean skin temperature ($\overline{T}$_sk_) was calculated from the seven points on the body using a method devised by Hardy and DuBois [29]. The diameter of the blood vessels (DBVs) at the second knuckle of the right middle finger was measured at 5 min intervals during the experiment using near-infrared spectroscopic imaging (Astrim, Sysmex Corp, Japan; Fig. 1). A near-infrared ray from a light emitting diode was passed through the blood vessels of a finger to hit the lens of a charge-coupled device camera, images from which DBV was calculated (Fig. 1) [22]. Oxygen consumption ($\overset{\cdot }{\mathrm{V}}$O_2_) was measured in a breath-by-breath manner (AE-300S, Minato Medical Science, Japan) during cold exposure.

Temperature sensors were fixed inside the rectum and at seven points on the skin, then a probe unit to measure DBV was fixed on the middle finger of the right hand. The mask for sampling expired gas was fixed on the mouth and nose, and then the participants rested in the supine position for at least 60 min in a room with ambient temperature and relative humidity maintained at 28 °C and 50%, respectively. Thereafter, the participants rested in the supine position for 90 min in a climatic chamber where the temperature and relative humidity were maintained at 10 °C and 50%, respectively. The subjects wore a short-sleeved cotton T-shirt and short cotton pants (0.3 clo).

After the experiment, percent of minimum DBV relative to the baseline (%DBV) was calculated as the minimum value divided by the baseline value [%DBV = (minimum DBV)/(baseline DBV) × 100]. The rate of vasoconstriction (%VC), percent change relative to the baseline, was also calculated using the equation as follows: %VC = 100 − %DBV. $\overset{\cdot }{\mathrm{V}}$O_2_ was used as an index of metabolic heat production [30], and the change in $\overset{\cdot }{\mathrm{V}}$O_2_ (%$\overset{\cdot }{\mathrm{V}}$O_2_) was calculated as the value at 90 min divided by the baseline value (0 min). Skin heat conductance (K_b_, W/m^2^°C) was calculated from the following equation [11, 12, 18]:

$$ {K}_{\mathrm{b}}\kern0.5em =\kern0.5em \left(R\kern0.5em +\kern0.5em C\right)/\left(\ {T}_{\mathrm{re}}\kern0.5em \hbox{--} \kern0.5em {\overline{T}}_{\mathrm{sk}}\right), $$

where (*R* + *C*) indicates radiant and convective heat exchange (in W/m^2^), calculated as (*R* + *C*) = *h* ($\overline{T}$_sk_ − *T*_db_), where *h* is the combined heat transfer coefficient for radiation and convection with a value of 8.3 W/m^2^ °C, that was determined as described by Colin et al. [31]. *T*_re_, $\overline{T}$_sk_, and *T*_db_ are rectal, mean skin, and dry bulb (10 °C) temperatures, respectively.

### Physical fitness test

Individual maximum oxygen consumption ($\overset{\cdot }{\mathrm{V}}$O_2max_) as a direct index of aerobic physical fitness was measured on the day after the cold exposure test to estimate the relationship between physical fitness and the observed findings. Oxygen consumption ($\overset{\cdot }{\mathrm{V}}$O_2_) was calculated in a breath-by-breath manner by ventilation and differences in oxygen concentrations of inspired and expired gases (AE-300S, Minato Medical Science, Japan) during physical exercise on a bicycle ergometer with a continuously incremental workload (+ 10 W/min). The test was terminated upon self-determined exhaustion or when the participant could no longer maintain the 50 rpm cadence. The criteria for achieving $\overset{\cdot }{\mathrm{V}}$O_2max_ included a respiratory gas exchange ratio exceeding 1.0 and visible signs of exhaustion, such as breathlessness and inability to maintain the required power output [12]. In this manner, the relationship between aerobic capacity and physiological responses to cold was estimated.

### Statistical analysis

The body temperature of one participant could not be recorded due to faulty sensors, and the DBV of another could not be recorded because the veins in the fingers were undetectable. Therefore, we analyzed the body temperature and DBV for six participants.

The average value of the last 5 min at 28 °C was used as the baseline value, and the average value for 88 to 90 min during cold exposure was used as the value at 90 min. The volume (∆) and rate of change (%) from the baseline value were also calculated, and some data were converted into logarithms. The measured value, ∆*T*_rec_, ∆$\overline{T}$_sk_, and %VO_2_ were used for statistical analysis.

In terms of rectal and skin temperatures, data at 0 and 90 min of cold exposure were analyzed by paired *t* test. Correlations between temperature decreases at the back of the hand (∆*T*_bh_) and in the rectal temperature (∆*T*_re_) compared with the respective baseline temperatures were analyzed using simple linear regression. Correlations between physical characteristics ($\overset{\cdot }{\mathrm{V}}$O_2max_, %fat, lean body mass (LBM), BSA, and subcutaneous fat thickness) and %$\overset{\cdot }{\mathrm{V}}$O_2_, *K*_b_, and %VC, as well as between %$\overset{\cdot }{\mathrm{V}}$O_2_ and %VC at the end of exposure to cold, were also analyzed using simple linear regression. *P* values below 0.05 were regarded as statistically significant.

## Results

*T*_re_ and temperatures at all skin locations reached a steady state after resting for 60 min in a thermo-neutral room. *T*_re_ of the two participants remained higher after cold exposure compared with before exposure, whereas in the other subjects, *T*_re_ decreased. All skin temperatures continuously decreased during cold exposure without stability. Shivering and/or goosebumps developed in all participants exposed to cold, and they reported feeling “very cold” thereafter.

Table 2 indicates the mean ± SD of *T*_re_, $\overline{T}$_sk_, all skin temperatures before and after cold exposure, and of changes in *T*_re_, $\overline{T}$_sk_, and all skin temperatures after cold exposure. The decreases in *T*_re_ were 0.18 ± 0.27 °C, which was not significant. Skin temperatures of each site and $\overline{T}$_sk_ were significantly decreased by 90-min cold exposure. The decreases in $\overline{T}$_sk_ were 7.89 ± 0.66 °C. Decreases in the forehead and abdominal (trunk region) skin temperatures were 5.32 ± 1.15 and 3.44 ± 0.33 °C, respectively, which were smaller than those at other sites of the skin. Skin temperature of the forearm, back of the hand, shin, and instep (peripheral sites) was markedly decreased by cold exposure, which was more than 10 °C (Table 2).

**Table 2:** Rectal and skin temperatures before and after cold exposure for 90 min

Site	0 min	90 min	Change	*P* value
Rectal temperature	37.01 ± 0.24	36.83 ± 0.46	0.18 ± 0.27	0.1794
Skin temperature				
Forehead	35.04 ± 0.23	29.70 ± 1.18	5.32 ± 1.15	< 0.0001
Abdomen	35.23 ± 0.65	31.79 ± 0.89	3.44 ± 0.33	< 0.0001
Forearm	33.62 ± 0.33	20.58 ± 1.97	13.05 ± 1.97	< 0.0001
Hand	34.59 ± 0.38	18.43 ± 1.08	16.16 ± 0.83	< 0.0001
Thigh	34.55 ± 0.86	27.63 ± 1.17	6.92 ± 1.15	< 0.0001
Shin	34.18 ± 0.42	24.13 ± 1.47	10.05 ± 1.40	< 0.0001
Instep	33.88 ± 0.83	18.84 ± 0.94	15.03 ± 0.68	< 0.0001
Mean *T*_sk_	34.60 ± 0.52	26.71 ± 0.93	7.89 ± 0.66	< 0.0001

Values are indicated as mean ± SD. Mean *T*_sk_ shows mean skin temperature calculated using Hardy and DuBois formula. *P* values were analyzed by paired *t* test

**Fig. 2:**
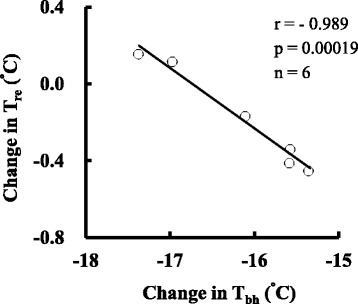
Relationship between change in temperature of the skin on the back of the hand and change in rectal temperature after 90 min of cold exposure. *T*_re_ and *T*_bh_ indicate rectal and back of the hand temperatures, respectively

Figure 2 shows the relationship between ∆*T*_bh_ that declined the most among the skin sites and ∆*T*_re_. The correlation between ∆*T*_bh_ and ∆*T*_re_ was significantly negative (*r* = − 0.989, *P* < 0.001) at the end of the cold exposure. The participants whose rectal temperatures remained higher in the cold had the lower skin temperatures at peripheral sites, such as the back of the hand.

**Fig. 3:**
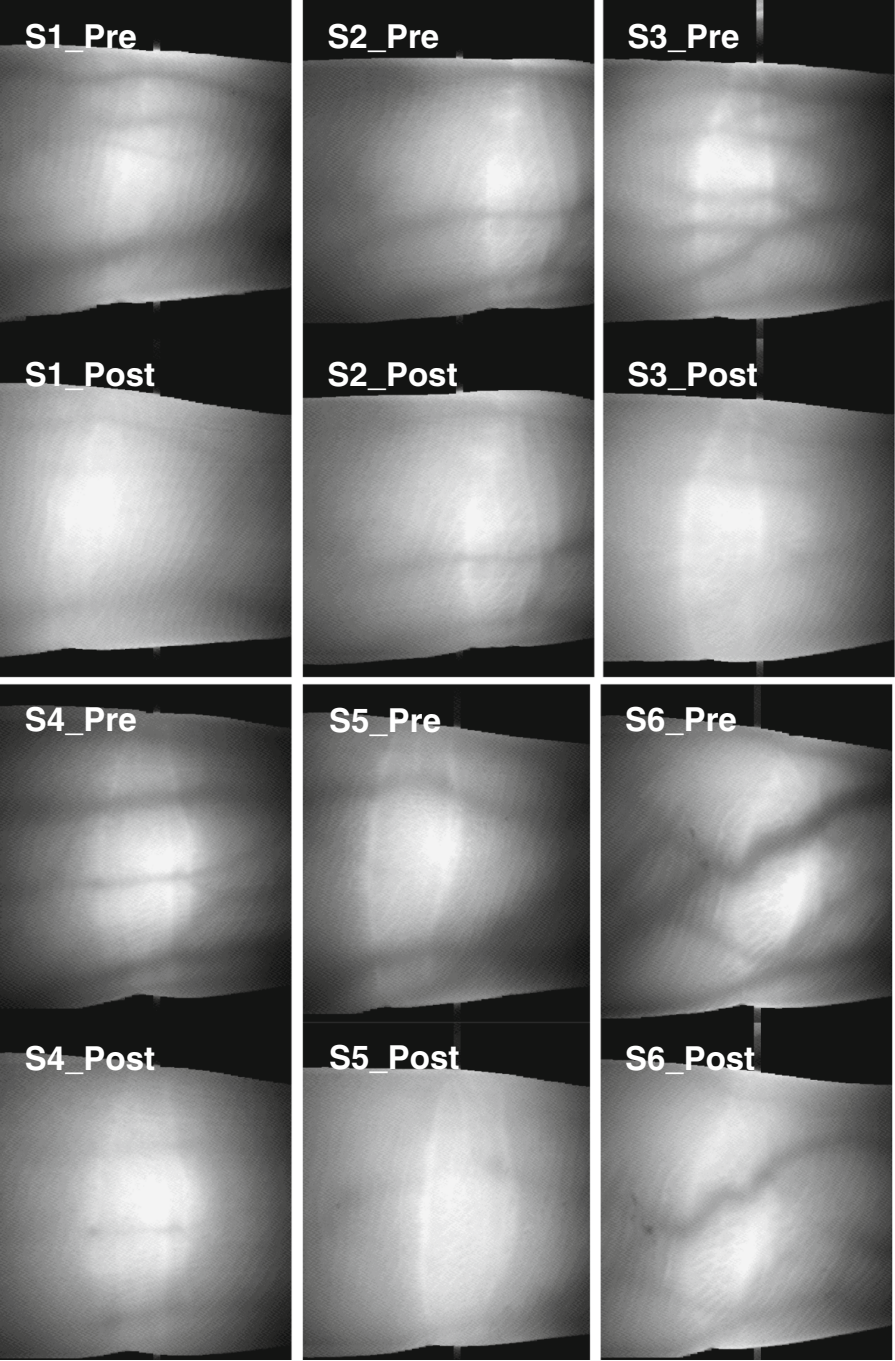
Near-infrared transmission image of light in the fingers before and after exposure to cold for 90 min

Figure 3 shows the transmission images of near-infrared light through the fingers before and after cold exposure for 90 min. The diameter of the blood vessels in all subjects was decreased by cold exposure for 90 min (baseline 7.5 ± 1.6 mm, 90 min 5.2 ± 1.7 mm, *P* < 0.01).

**Fig. 4:**
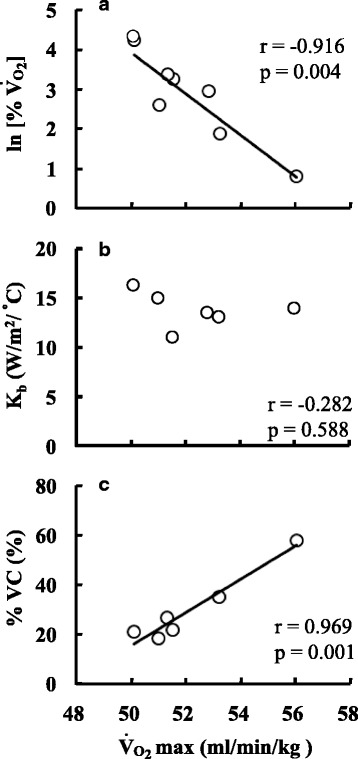
Relationships between maximum oxygen consumption ($\overset{\cdot }{\mathrm{V}}$O_2max_) and (**a**) the increase in oxygen consumption (%$\overset{\cdot }{\mathrm{V}}$O_2_), (**b**) skin heat conductance (*K*_b_), and (**c**) percent of vasoconstriction (%VC) after 90 min exposure to cold

Figure 4 shows the relationships between $\overset{\cdot }{\mathrm{V}}$O_2max_ and the % change in $\overset{\cdot }{\mathrm{V}}$O_2_ (Fig. 4a), *K*_b_ (Fig. 4b), and the % VC (Fig. 4c) at 90 min during cold exposure. The correlation between $\overset{\cdot }{\mathrm{V}}$O_2max_ and %$\overset{\cdot }{\mathrm{V}}$O_2_ at 90 min cold exposure was significantly negative (*r* = − 0.916, *P* = 0.004). Metabolic heat production in response to cold exposure increased less in a fitter than in a less-fit individual (Fig. 4a). The correlation between $\overset{\cdot }{\mathrm{V}}$O_2max_ and K_b_ at 90 min during cold exposure was not significant (Fig. 4b, *r* = − 0.283, *P* = 0.586). With respect to the degree of vasoconstriction, although $\overset{\cdot }{\mathrm{V}}$O_2max_ and DBV did not correlate, $\overset{\cdot }{\mathrm{V}}$O_2max_ and %VC (*r* = 0.954, *P* = 0.003) calculated from the change in DBV were significantly and positively correlated (Fig. 4c). Thus, the peripheral blood vessels were more constricted during cold exposure in fitter than in less-fit individuals.

**Fig. 5:**
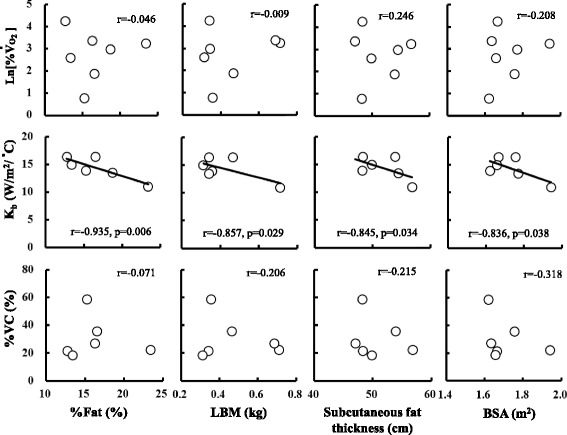
Relationships between %fat, lean body mass (LBM), subcutaneous fat thickness, body surface area (BSA), change in increasing rate of oxygen uptake (%$\overset{\cdot }{\mathrm{V}}$O_2_), skin heat conductance (*K*_b_), and rate of vasoconstriction (%VC) after 90 min of cold exposure

Figure 5 shows the relationships between physical characteristics as %fat, LBM, subcutaneous fat thickness, and BSA and ln[%$\overset{\cdot }{\mathrm{V}}$O_2_] (four upper panels), *K*_b_ (four middle panels), and %VC (four bottom panels) at 90 min of cold exposure. Physical characteristics (%fat, LBM, BSA, and subcutaneous fat thickness) did not influence either metabolic heat production (%$\overset{\cdot }{\mathrm{V}}$O_2_) or peripheral vasoconstriction (%VC). However, *K*_b_ was significantly affected by %fat (*r* = − 0.936, *P* = 0.006), LBM (*r* = − 0.857, *P* = 0.029), BSA (*r* = − 0.836, *P* = 0.038) and subcutaneous fat thickness (*r* = − 0.841, *P* = 0.036).

**Fig. 6:**
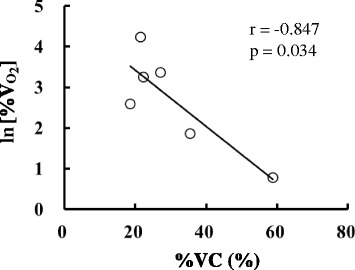
Relationship between the rate of vasoconstriction (%VC) and the increase in oxygen uptake (%$\overset{\cdot }{\mathrm{V}}$O_2_)

Figure 6 shows the relationship between %VC and logarithms of the increase in $\overset{\cdot }{\mathrm{V}}$O_2_. There were negative relationships between %VC and ln[%$\overset{\cdot }{\mathrm{V}}$O_2_] (*r* = − 0.847, *P* = 0.034). Metabolic heat production was not increased in participants whose peripheral blood vessels were more constricted during cold exposure. Instead, metabolic heat production was increased in those whose blood vessels were less constricted.

## Discussion

This study found that (1) core temperature was better maintained in individuals with relatively low peripheral skin temperature, (2) vasoconstriction of the finger veins was more pronounced in individuals with higher maximum oxygen uptake than in others with lower maximum oxygen uptake, and that (3) metabolic heat production increased more from the baseline in individuals whose finger blood vessels were less constricted during cold exposure.

Peripheral vasoconstriction is important for thermoregulation in a cold environment, because the decrease in peripheral skin temperature, controlled by vasoconstriction, suppresses heat loss from the body surface and then the core temperature is better maintained. High ability of peripheral vasoconstriction enables the regulation of body temperature without the metabolic thermogenesis that is the second stage of the thermoregulation after the vasoconstriction.

Stromme and Hammel (1967) found that physically active rats produced more metabolic heat in a cold environment than inactive rats [32]. Bittel et al. (1988) also found a significant positive correlation between physical fitness and levels of metabolic heat production and skin heat conductance induced by acute exposure to cold at 1, 5, and 10 °C for 2 h in human [11]. These results showed that body temperature was not regulated only by suppression of heat loss caused by the vasoconstriction which was the first stage of thermoregulation; metabolic heat production which was the second stage occurred in not only inactive rat or lower fit person but also in active rat or higher fit person. It is thought that the increase of the metabolism to bring thermogenesis promotes movement of the heat to the skin, and then the heat loss increased. The results of the present study contradict these results in terms of metabolic heat production that negatively correlated with maximum oxygen uptake and skin heat conductance that did not correlate with maximum oxygen uptake. Maeda et al. (2007) indicated that metabolic heat production of individuals with a high basal metabolic rate, which was closely correlated with resting metabolic rate and muscle mass, was not increased during cold exposure because their core temperatures were only regulated by suppressing heat loss from the body surface [33]. The results of the present study might be influenced by not only the ability of vasoconstriction but also basal metabolic rate because it was though there was a relationship between maximum oxygen uptake and basal metabolic rate.

On the other hand, since individuals who are very fit also have more muscle mass, that is the main source of heat generation through shivering, the potential for heat production is high, and sufficient heat can be produced on demand to regulate core temperature. Accordingly, the present and previous results differ because the stages of thermoregulation were different. However, this raises the question of why the stages of the thermoregulation were different. Some explanations are that the actual living environment influences the acclimation of individuals, the contents of meals influence blood vessel compliance and/or heat production, and/or somatotype influences the ability to lose or generate heat. Body and subcutaneous fat might also be factors.

Bittel et al. (1988) indicated that the ratio of body fat significantly correlates with metabolic heat production and mean skin temperature [11]. In addition, Budd et al. (1991) also associated fat proportion with reduced heat production and heat loss, although physical fitness had no effect on heat production and heat loss during cold exposure [20]. However, Yoshida et al. (1998) reported that skin heat conductance was lower in the trained, than in untrained, individuals with the same body fat content [18]. The present study found that %fat and subcutaneous fat thickness were negatively related to skin heat conductance but unrelated to metabolic heat production and vasoconstriction. Body fat, especially subcutaneous fat, plays a role in the suppression of heat loss from internal to external milieus. Therefore, the negative correlation between subcutaneous fat and *K*_b_, which is an index of heat loss, is reasonable, and the results of this study agreed with those of a previous investigation [11, 20]. On the other hand, our findings of metabolic heat production did not agree with published results. Since an increase in metabolic heat production depends mainly on shivering of the muscle and not fat, body fat should not be related to metabolic heat generation. Previous results that have found a negative correlation between body fat and metabolic heat production might be caused by suppressing heat loss by body fat. However, these results might also include the influence of less ability of heat production, because persons with more fat might have less muscle, which would decrease metabolic heat production. In the present study, the body fat content and subcutaneous fat thickness were 16.6 ± 3.60% and 0.50 ± 0.17 cm, respectively, values that were within the normal range for young adult Japanese males. So it was thought that the effect of body fat on cold-induced metabolic heat production is very small in the present study which was the normal somatotype and narrow range of body fat variation of our study participants.

Most of the previous studies have estimated vasoconstriction under cold conditions only by measuring skin temperature at several sites, skin blood flow, and/or skin heat conductance, calculated using core, skin, and ambient temperatures. Here, we directly observed vasoconstriction in response to cold using near-infrared imaging of the finger blood vessels and found a significant positive correlation between maximum oxygen uptake and vasoconstriction. These results suggested that vasoconstriction ability is a sensitive index for evaluating cold tolerance.

The finding of greater vasoconstriction in fitter persons suggests increased sensitivity of the control of vasoconstriction to cold stimuli among such individuals. The mechanisms of physiological change-related thermoregulation associated with aerobic exercise are the improvements in compliance of the peripheral blood vessels [23–26], autonomic nervous function controlling the vasomotor system [27, 28], and vasomotor responses to thermal stimulation [34].

## Conclusion

In conclusion, the present results suggested that the capacity for peripheral vasoconstriction is improved by physical exercise and that physical fitness is associated with enhanced ability to maintain metabolic heat production in a cold environment at resting metabolic levels of a thermoneutral condition. Fit individuals appear to have a greater capacity for vasoconstriction when exposed to cold and thus also appear to lose less heat than their less-fit counterparts.

## Abbreviations

%DBV: Rate of change in diameter of the blood vessels
%$\overset{\cdot }{\mathrm{V}}$O_2_: Increase rate of oxygen consumption
(*R* + *C*): Radiant and convective heat exchange
∆*T*_bh_: Change in skin temperature at the back of the hand
BSA: Body surface area
DBV: Diameter of the blood vessels
*K*_b_: Skin heat conductance
LBM: Lean body mass
$$ \overset{\cdot }{\mathrm{V}} $$O_2_: Oxygen consumption
$\overset{\cdot }{\mathrm{V}}$O_2max_: Maximum oxygen consumption
$\overline{T}$_sk_: Mean skin temperature
*T*_re_: Rectal temperatures
